# TnT: a set of libraries for visualizing trees and track-based annotations for the web

**DOI:** 10.1093/bioinformatics/btw210

**Published:** 2016-04-22

**Authors:** Miguel Pignatelli

**Affiliations:** Centre for Therapeutic Target Validation and European Bioinformatics Institute, Wellcome Genome Campus, Hinxton, Cambridge CB10 1SD, UK

## Abstract

**Summary:** There is an increasing need for rich and dynamic biological data visualizations in bioinformatic web applications. New standards in web technologies, like SVG or Canvas, are now supported by most modern web browsers allowing the blossoming of powerful visualizations in biological data analysis. The exploration of different ways to visualize genomic data is still challenging due to the lack of flexible tools to develop them. Here, I present a set of libraries aimed at creating powerful tree- and track-based visualizations for the web. Its modularity and rich API facilitate the development of many different visualizations ranging from simple species trees to complex visualizations comprising per-node data annotations or even simple genome browsers.

**Availability and Implementation:** The TnT libraries have been written in Javascript, licensed under the APACHE 2.0 license and hosted at https://github.com/tntvis.

**Contact:** mp@ebi.ac.uk

## 1 Introduction

The web has become the preferred platform to present biological data to the scientific community. Several improvements in web technologies are enabling the blossoming of new web applications for visualising this data: First, support of well established standards like SVG and Canvas by all modern web browsers enables the creation of rich interactive visual displays. Second, the vast improvement in web browser performance has increased the complexity of visualizations. Third, the proliferation of RESTful services providing data from reference biological resources permits easy access to biological data directly from the browser. The direct availability of this data also facilitates the development of reusable visualizations embeddable directly in web pages.

Reusability is another key component in modern web development and a strong trend in current biological visualizations. BioJS (Gómez *et al.*, 2013) aims to compile reusable widgets for biological data visualization. It compiles over 120 components at the time of writing (January 2016). However, writing reusable visualizations is a complex task in which the developer has to take into account the *scope* and the *environment* of the host application in which the visualization will be embedded.

Representation of tree data structures has become very popular in different biological fields like phylogenetics or ontology visualization, while the representation of track-based annotations is central to genome browsers (Kent *et al.*, 2002; Yates *et al.*, 2016), sequence alignments (Larsson, 2014; Waterhouse *et al.*, 2009) or general display of coordinate-based features such as protein sequence domain (Finn *et al.*, 2014).

Here, a new set of libraries aimed at creating configurable, dynamic and interactive re-usable visualizations of trees and track-based annotations is presented. They are collectively called *TnT*, standing for *Trees and Tracks*, and are distributed as independent *npm* packages for easier integration in web applications. The development of the TnT libraries have been driven by two main design principles: *flexibility* and *reusability*. Flexibility is achieved by exposing all its internals in a powerful and carefully designed API. Reusability is maximized by developing the libraries as independent units that can be combined.

TnT is being used by Ensembl (Yates *et al.*, 2016) to display comparative genomics annotations and the Centre for Therapeutic Target Validation (http://www.targetvalidation.org) to display both genomic features and tree-based annotation in their websites.

## 2 Results

**Fig. 1.**
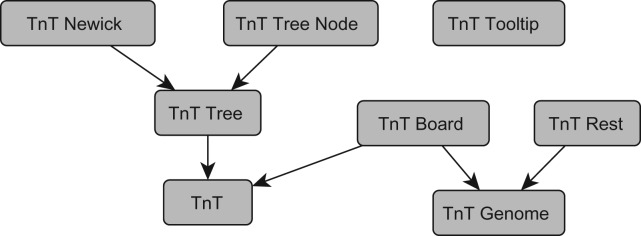
Interdependency graph showing the relationship between the different TnT libraries

The TnT libraries have been written in Javascript using the D3 library (http://d3js.org) as its main dependency. It uses SVG to render all the visual elements in the browser. Figure 1 shows the interdependency graph of the different libraries. A short description of each one follows:

### 2.1 TnT Tree

This library is built on top of the *D3 cluster* layout and allows building dynamic and interactive trees for the web. It is composed of several configurable elements: a *layout* that defines the general shape of the tree. Tree *nodes* that can be configured independently in shape, size and color. *Labels* composed of text or images and *data* for loading Javascript objects or newick/nhx strings. PhyloCanvas (http://phylocanvas.org) is a similar project offering reusable and dynamic tree visualizations. It uses Canvas as its main technology and offers a rich API. TnT Tree versatility and integration with other TnT libraries (see below) are distinctive features not available in similar libraries. Documentation and examples for TnT Tree can be found at http://tntvis.github.io/tnt.tree/.

### 2.2 TnT Tree Node

This library provides methods for tree manipulation at the data level and is used by TnT Tree although it can be used independently for manipulating tree-like hierarchical structures. The methods included in TnT Tree Node range from computing the lowest common ancestor of a set of nodes to extracting subtrees. The documentation for this library can be found as part of the TnT Tree library documentation.

### 2.3 TnT Board

This library facilitates the creation and configuration of track-based visualizations. A board is an interactive container for tracks in which each panning and zooming event triggers new data and visualization updates. Separation of concerns between data and visualization updates is one of its main features. The library FeatureViewer (Garcia et al., 2014) offers similar functionalities for displaying UniProt data (Consortium, 2015) without the flexibility offered by TnT Board regarding data updates and visual representation. Documentation of TnT Board can be found at http://tntvis.github.io/tnt.board/.

### 2.4 TnT Genome

TnT Genome is a simple genome browser library built on top of TnT Board. TnT Genome exposes some additional elements to facilitate the creation of custom-made simple genome browsers like retrieving Ensembl data through its REST API (Yates *et al.*, 2015), visual representation of genes and transcripts and avoiding overlaps between these elements. There are many interactive, re-usable web-compatible genome browsers already available like Genoverse (http://www.genoverse.org), Genome Maps (Medina *et al.*, 2013) or Biodalliance (Down *et al.*, 2011). Compared to them, TnT Genome presents a more flexible lower level library to create custom-tailored, simple genome browsers. Documentation for this library can be found at http://tntvis.github.io/tnt.genome/

### 2.5 TnT Rest

A general Javascript library to interface with RESTful services. It is based on Promises and is used by TnT Genome to retrieve Ensembl data via its RESTful API (Yates *et al.*, 2015).

### 2.6 TnT

TnT bundles together TnT Tree and TnT Board and connects both allowing per-node annotation tracks. This library facilitates the creation of annotated trees like gene trees in Ensembl (Yates *et al.*, 2016) or Wasabi (Veidenberg *et al.*, 2015). Documentation for this library can be found at http://tntvis.github.io/tnt/.

In summary, the TnT set of libraries offer a flexible way to create re-usable visualizations for the web in an integrated way. Being independent libraries yet able to interoperate is one of the main benefits over other available options.

## References

[btw210-B1] Consortium T.U. (2015) UniProt: a hub for protein information. Nucleic Acids Res., 43, D204–D212. 25348405 10.1093/nar/gku989 PMC4384041

[btw210-B2] Down T.A (2011) Dalliance: interactive genome viewing on the web. Bioinformatics, 27, 889–890. 21252075 10.1093/bioinformatics/btr020 PMC3051325

[btw210-B3] Finn R.D (2014) Pfam: the protein families database. Nucleic Acids Res., 42, D222–D230. 24288371 10.1093/nar/gkt1223 PMC3965110

[btw210-B4] Garcia L (2014) FeatureViewer, a BioJS component for visualization of position-based annotations in protein sequences. F1000Research, 3, 47. 24741440 10.12688/f1000research.3-47.v1 PMC3983936

[btw210-B5] Gómez J (2013) BioJS: an open source JavaScript framework for biological data visualization. Bioinformatics, 29, 1103–1104. 23435069 10.1093/bioinformatics/btt100 PMC3624812

[btw210-B6] Kent W.J (2002) The human genome browser at UCSC. Genome Res., 12, 996–1006. 12045153 10.1101/gr.229102 PMC186604

[btw210-B7] Larsson A. (2014) AliView: a fast and lightweight alignment viewer and editor for large datasets. Bioinf. Oxf. Engl., 30, 3276–3278. 10.1093/bioinformatics/btu531 PMC4221126 25095880

[btw210-B8] Medina I (2013) Genome Maps, a new generation genome browser. Nucleic Acids Res., 41, W41–W46. 23748955 10.1093/nar/gkt530 PMC3692043

[btw210-B9] Veidenberg A (2015) Wasabi: an integrated platform for evolutionary sequence analysis and data visualization. Mol. Biol. Evol., msv333. 10.1093/molbev/msv333 26635364

[btw210-B10] Waterhouse A.M (2009) Jalview Version 2–a multiple sequence alignment editor and analysis workbench. Bioinf. Oxf. Engl, 25, 1189–1191. 10.1093/bioinformatics/btp033 PMC2672624 19151095

[btw210-B11] Yates A (2015) The Ensembl REST API: ensembl data for any language. Bioinforma. Oxf. Engl., 31, 143–145. 10.1093/bioinformatics/btu613 PMC4271150 25236461

[btw210-B12] Yates A (2016) Ensembl 2016. Nucleic Acids Res., 44, D710–D716. 26687719 10.1093/nar/gkv1157 PMC4702834

